# Forecasting fluid-injection induced seismicity to choose the best injection strategy for safety and efficiency

**DOI:** 10.1098/rsta.2023.0179

**Published:** 2024-08-09

**Authors:** Auregan Boyet, Víctor Vilarrasa, Jonny Rutqvist, Silvia De Simone

**Affiliations:** ^1^ Global Change Research Group (GCRG), IMEDEA, CSIC-UIB, Esporles, Spain; ^2^ Associated Unit: Hydrogeology Group (UPC-CSIC), Barcelona, Spain; ^3^ Lawrence Berkeley National Laboratory, Berkeley, CA, USA; ^4^ Institute of Environmental Assessment and Water Research, Spanish National Research Council (IDAEA-CSIC), Barcelona, Spain

**Keywords:** induced seismicity, enhanced geothermal system

## Abstract

Induced seismicity poses a challenge to the development of Enhanced Geothermal Systems (EGS). Improving monitoring and forecasting techniques is essential to mitigate induced seismicity and thereby fostering a positive perception of EGS projects among local authorities and population. Induced seismicity is the result of complex and coupled thermo-hydro-mechanical-chemical mechanisms. Injection flux and pressure are crucial controlling parameters for both hydraulic stimulation and circulation protocols. We develop a methodology combining a hydro-mechanical model with a seismicity rate model to estimate the magnitude and frequency of mainshocks and aftershocks induced by fluid injection. We apply the methodology to the case of the Basel EGS (2006, Switzerland) to compare the effects of progressive, cyclic and constant injections on the mechanical response of discrete faults. Results from the coupled hydro-mechanical models show that the pore pressure diffusion and consequent enhancement of fault permeability are limited to the vicinity of the injection well during cyclic injection. Additionally, constant injection induces seismicity from the start of the injection but enhances the permeability of most of the faults within a shorter duration, inducing less post-injection seismicity. The methodology can be adapted to any numerical model and allows new projects to be developed by anticipating the safest injection protocol.

This article is part of the theme issue ‘Induced seismicity in coupled subsurface systems’.

## 1. Introduction

Low-carbon emission geo-applications are a major instrument in the reduction of carbon emissions to reach the 2050-IPCC goals [1]. Current investigations focus on the development of geological carbon and hydrogen storage and geothermal energy projects. The principal obstacles to these applications reside in the hydraulic constraints of the geological reservoir and subsequent induced seismicity. Fluid injection and extraction disturb the effective stress field in the subsurface, leading to the reactivation of pre-existing faults and to the creation of new ones. Each geo-energy application has its characteristics and limitations, depending on the thermal and geochemical interactions between the injected fluid, the rock and the fluids initially present in the rock. Enhanced Geothermal Systems (EGS) aim to produce electricity by exploiting the heat from the deep and hot crystalline basement. Because of the low permeability of the rock, the reservoirs are engineered to enhance the permeability of the natural fractures, to improve fluid circulation between the injection and the production wells, via hydraulic or acid stimulations [2]. This technology has been developed since the 1980s in many locations in the world [3–7]. Yet, economic viability and public perception hinder the exploitation of new EGS projects. Induced seismicity is expected during hydraulic stimulation and production, with a magnitude generally lower than $M_{w}2.0$ . Nevertheless, larger-magnitude induced seismicity has been felt by local populations in different cases of EGS. Probably the most controversial and studied case is the Basel Deep Heat Mining project in Switzerland, 2006, where after only six days of injection (11 570 m^3^ of water at 4630 m depth), the magnitude of the induced seismicity reached the threshold of the traffic light system ( $M_{w}2.3$ ). The injection was consequently stopped, but five hours later the largest-magnitude earthquake occurred ( $M_{l}3.4-M_{w}2.9$ ) and led to the decision of bleeding-off the well [8]. Perceivable induced seismicity kept occurring for months after the cancellation of the project. The recent case of Pohang EGS, South Korea, in 2017 further questioned the safety of EGS in urban areas. A $M_{w}5.4$ earthquake occurred two months after the stop of injection and caused damages in the surrounding urban areas, leading to the cancellation of the project [9–11]. More recently, in 2021, a $M_{l}3.9$ earthquake occurred six months after the stop of a circulation test in Vendenheim, France, and led to the cancellation of the EGS project [12–14].

The triggering mechanisms of induced seismicity are difficult to apprehend, and their coupling makes it difficult to model and forecast [5,15,16]. Fluid injection increases the pressure in the reservoir, which induces poromechanical deformations and stress changes. These pressure-driven processes affect the hydro-mechanical properties of the rock and faults. Fault reactivation and fracture propagation modify the permeability of the rock, consequently affecting the fluid flow. Fault slips, which can be aseismic or seismic, can also further destabilize the reservoir due to processes of static stress transfer [17–19]. These triggering mechanisms continue to occur after the stop of injection. Pressure-driven processes continue to diffuse for a long duration, while poroelasticity and static stress transfer continue to occur for a shorter time scale [20].

Because all these processes are initiated by fluid injection, they are affected by the injection protocol, which can vary in terms of applied pressure, injection rate and duration. New strategies are investigated to mitigate the frequency and magnitude of earthquake nucleation. For example, cyclic injection is currently seen as a promising solution to enhance the permeability of EGS reservoirs while controlling the seismicity based on the concept of fatigue hydraulic fracturing, as shown by numerical studies [21,22] and validated in rock laboratory experiments [23–25]. In contrast, Noël *et al*. [26] showed that the stable slip from a displacement-driven fault can become unstable and induce seismicity during cyclic injection. Cyclic injection was also applied during the EGS projects of Pohang, South Korea [27] and of Helsinki, Finland [28]. In the Pohang EGS, the co-injection magnitude threshold of $M_{w}2.0$ was never reached, but the largest-magnitude ( $M_{w}5.4$ ) earthquake ever induced in an EGS occurred two months after the stop of the fifth stimulation [9–11]. The Helsinki EGS project also successfully controlled the induced seismicity under the magnitude threshold set by the local authorities, but permeability enhancement was insufficient [28]. Hydraulic fatigue in cyclic injection is also considered as a mitigation injection strategy, by inducing microcracks to avoid large failures [29,30]. Strategies for stopping injection are also investigated from the perspective of controlling the post-injection induced seismicity that occasionally occurs with a larger magnitude than the co-injection seismicity. For example, a progressive stop of injection has been proposed to mitigate post-injection induced seismicity [31,32]. Effectively, a progressive decrease of injection lessens the effects of pore pressure changes and mitigates the destabilizations of the early post-injection stage [33].

Currently, numerical models are developed to understand, simulate and forecast fluid-injection induced seismicity. Numerical models, also called physics-based models, are the best tool to solve coupled problems of pressure-driven, thermal, geochemical and geomechanical processes due to fluid injection and extraction on the subsurface. These models require a characterization of the reservoir, including rock properties, *in situ* pressure and stress conditions and geological settings, which are difficult data to measure. Analytical and statistical models make up for the lack of data by applying different statistical laws to the observed problems. Many statistical models have been developed to forecast seismicity (e.g. [34,35]). For instance, the Seismogenic Index [36–38] forecasts the induced seismicity rate mainly as a function of the injected volume [39,40]. Hybrid models actually combine physics-based models with statistical laws to develop robust tools to forecast induced seismicity. Different methodologies have been proposed, building on seed models (e.g. [41–43]), or based on discrete faulting models (e.g. [44,45]). All models have their strengths and weaknesses: seed models have the advantage of providing fast computations but they lack representation of the physical and mechanical processes, while discrete models solve more complex problems, but with a high computational cost to be used as an efficient forecasting tool.

Here, we propose a hybrid methodology based on a hydro-mechanical model of a discrete fault network associated with a seismicity rate model and statistical seismological laws. We apply the methodology to the case of the Deep Heat Mining Project in Basel, Switzerland. Induced seismicity in Basel EGS has been intensively studied in the last two decades. Seismic interpretations provide a rich and accurate catalogue of earthquakes [46–48]. Pore pressure buildup was suggested as the triggering mechanism of the induced seismicity due to the short-duration injection, but this assumption is insufficient to explain the nucleation of earthquakes far from the injection well and shortly after the stop of injection [49,50]. Mukuhira *et al*. [51] explained this far post-injection seismicity to be induced by means of the homogenization of pore pressure buildup along a large fault when the injection was stopped. The diffusion of the pore pressure would have reached the critical pressure along the fault plane. Moreover, the triggering effects of static stress transfer caused by nucleation stress drop were measured and defined as partly triggering the post-injection induced seismicity [52,53]. This latter study proposed a decoupling of a hydraulic model with the stress drop calculation. A few coupled models with discrete faults propose more convincing explanations concerning the fluid-injection effects on the induced seismicity in the Basel EGS. Andrés *et al* [54] provided a thermo-hydro-mechanical model of one large discrete fault, and Boyet *et al*. [33] used a hydro-mechanical model to study the reactivation of a fault network. The post-injection induced seismicity at Basel can be reproduced by the combination of the poroelastic relaxation due to the shut-in of injection with the static stress transfer of reactivating faults [33]. In this study, we adopt the same physics-based model of Boyet *et al*. [33], which simulates fault failure at the location and timing of the observed earthquakes at Basel, and we improve the hybrid method, estimating seismicity rate on a simple geometry model as proposed by Boyet *et al*. [20]. We analyze the response to different injection strategies and we forecast the best strategy in terms of permeability enhancement and inducing seismicity.

## 2. Hybrid method to forecast induced seismicity

**Figure 1.**
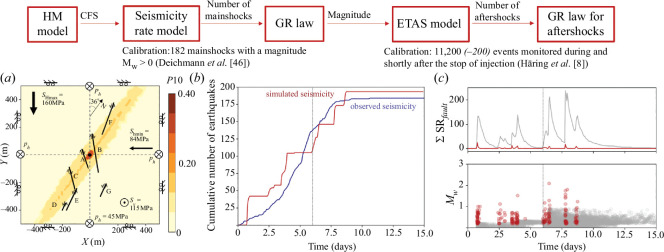
Hybrid methodology: the Coulomb failure stress (CFS) output from the hydro-mechanical (HM) model is the main input of the seismicity rate model. The GR law and the ETAS model estimate the magnitude and the aftershock sequence of the mainshocks. (*a*) Hydro-mechanical model with the fault network. Note that the *x*- and *y*-axes are not aligned with the north but with the principal stresses. The injection well is represented by the black dot in the centre. Colours refer to the probability of hosting a fracture, which is equal to 1 on faults A–G. (*b*) Temporal evolution of the cumulative number of observed mainshocks from the catalogue of Deichmann *et al*. [46] (blue line) and of simulated mainshocks in the injection scenario used for calibration, which reproduces the operations at Basel (injection during 6 days followed by 5 hour shut-in and a bleed-off until the end) (red line). (*c*) Time evolution of the number of mainshocks (red) and aftershocks (grey), and time evolution of the magnitude of seismic events in the domain during the calibration injection case.

The hybrid method is based on the combination of a hydro-mechanical model of a discrete fault network with a seismicity rate model (figure 1). The seismicity rate estimates the number of mainshocks occurring during the simulation as a function of the Coulomb stressing rate, calculated in the hydro-mechanical model. Their magnitude and aftershock sequence are then, respectively, estimated by the Gutenberg–Richter (GR) law [55] and the Epidemic Type Aftershock Sequence (ETAS) model [56]. The application of the methodology to the case of the Basel EGS aims to compare the seismicity induced by different injection protocols. Nevertheless, the proposed methodology could be also applied in ‘real time’ to forecast seismicity for further injection stages.

### (a) Hydro-mechanical model

The hydro-mechanical problem is solved by means of the finite-element method simulator CODE_BRIGHT [57], with the adoption of a continuum approach. Here, the model is limited to two dimensions due to the complexity of the discrete fault network and to the computation time. The hydro-mechanical problem is solved in a fully coupled way, solving the momentum balance and the water mass balance simultaneously. The former reads


(2.1) ∇⋅σ+b=0,

where **σ** is the stress tensor and **b** is the vector of body forces. The latter is expressed as


(2.2) $$\phi \beta \frac{\partial P}{\partial t}+\frac{\partial }{\partial t}\varepsilon_{v}+\nabla \cdot q=f^{w},$$

where ϕ is rock porosity, β is water compressibility, P is water pressure, $\varepsilon_{v}$ represents volumetric strain, t is time and $f^{w}$ is an external supply of water; q is the water volumetric flux and is expressed by Darcy’s law


(2.3) $$q=-\frac{k}{\gamma }\left(\nabla P-\rho g\right),$$

where γ and ρ are water viscosity and density, respectively, and g is the gravity vector. The intrinsic permeability k of the matrix is a function of porosity by means of Kozeny’s model as $k=k_{o}\frac{\phi^{3}}{{\left(1-\phi \right)}^{2}}\frac{{\left(1-\phi_{o}\right)}^{2}}{{\phi_{o}}^{3}}$ , with $k_{o}$ and $\phi_{o}$ being, respectively, the reference values for the intrinsic permeability and the porosity of the rock matrix. The permeability of the matrix is assumed to be isotropic. The coupling of the governing equation (2.1) and equation (2.2) is expressed through the elastic constitutive law of the matrix


(2.4) $$\Delta \sigma =K\varepsilon_{v}\;I+2G\left(\varepsilon -\frac{\varepsilon_{v}}{3}I\right)-\Delta PI,$$

where K=E/[3(1-2ν)] is the rock bulk modulus, G=E/[2(1+ν)] is the shear modulus, E is Young’s modulus, ν is Poisson’s ratio, ε is the strain tensor and I is the identity matrix. Note that the volumetric strain $\varepsilon_{v}$ is the first invariant of the strain tensor, i.e. $\varepsilon_{v}=tr(\varepsilon )$ . The effective stress law is enclosed in equation (2.4) with the Biot coefficient equal to 1.

On the other hand, the discrete faults are represented by finite-thickness elements following the ‘embedded model’ proposed by Olivella and Alonso [58]. In this conceptual model, faults are defined by their aperture embedded in a continuous finite element composed of rock matrix. Based on the cubic law, the permeability of these fault elements varies proportionally to the square of the fracture aperture, which depends on the volumetric strain of the fault elements. The volumetric strain accounts for both reversible (elastic) and irreversible (plastic) deformation. The initial minimum/maximum value of the permeability of fault elements is set based on the calibration of the model (table 1). Fault elements are subject to the Mohr–Coulomb failure model including dilatancy and their visco-plastic constitutive behaviour is modelled by the following equations (2.5)–(2.8)

**Table 1.** Material properties of matrix and fault elements of the hydro-mechanical model [33].

parameters	matrix	fault element
porosity, ϕ (−)	0.01	0.1
permeability, k (m^2^)	7.50 × 10^−17^	initial: 2.30 × 10^−13^ – 7.50×10^−13^
Young’s modulus, E (GPa)	52	43
Poisson’s ratio, ν (−)	0.25	0.25
cohesion	0	0
friction coefficient	0.6	0.35–0.58 (set with calibration)


(2.5) $$\frac{d\varepsilon_{p}}{dt}=\;\Gamma \left\langle \Phi \left(F\right)\right\rangle \frac{\partial \xi }{\partial \sigma },$$

where $\varepsilon_{p}$ is the visco-plastic strain, and Γ is the fluidity, a parameter of the deformation of the medium set at 10^−4^ MPa^−m^ s^−1^; $\Phi \left(F\right)$ is the overstress function, described in equation (7) and ξ is the flow rule, which reads


(2.6) $$\xi =\;\alpha \cdot \sigma_{m}\cdot \sin\psi +\left(\cos\theta -\frac{1}{\sqrt{3}}sin\theta \cdot \sin\psi \right)\cdot \sqrt{J_{2}}-c\cdot cos\psi ,$$

where α is a parameter for the plastic potential, set at 1, ψ is the dilatancy angle, set as 3°, $\sigma_{m}$ is the the mean stress, $J_{2}$ and θ (Lode’s angle) are invariants of the deviatoric stress tensor; c is the cohesion (set to zero in this model). The visco-plastic constitutive behaviour is estimated with the overstress function $\Phi \left(F\right)$ :


(2.7) $$\Phi \left(F\right)=\;\;\left\{ \begin{array}{l} 0,\;\;\;\;if\;F\leq 0\;\\ F^{m},\;\;\;if\;F>0\; \end{array}\right.,$$

where m is a constant power to define the overstress function (set as 3 in this model) and F is the yield function, which describes the conditions under which a material undergoes plastic deformation, defined as


(2.8) $$F=\;\sigma_{m}\cdot \sin\varphi \left(\eta \right)+\left(\cos\theta -\frac{1}{\sqrt{3}}sin\theta \cdot sin\varphi \right)\cdot \sqrt{J_{2}}-c\cdot cos\varphi ,$$

with φ the friction angle, calibrated for each fault to reproduce the timing of reactivation. The fault elements follow a viscoplastic constitutive behaviour. Their deformation is elastic until the stresses reach the shear yield surface, F=0 . During the elastic regime, a constitutive law similar to equation (2.4) holds. When the yield surface is exceeded, the fault element deforms irreversibly according to the viscoplastic behaviour, but stresses can remain beyond the yield surface for a range determined by the overstress function $\Phi \left(F\right)$ . The permeability enhancement mostly occurs when the fault element deforms plastically.

In this hydro-mechanical model, the effects of pressure-driven processes, i.e. pressure buildup and poroelasticity, are combined with the static stress transfer caused by fault reactivation. To measure the mechanical stability of discrete faults, the Coulomb Failure Stress ( CFS ) is calculated as


(2.9) $$CFS=\;\tau -\;\sigma_{n}^{\prime }\times \mu +c,$$

where τ and $\sigma_{n}^{\prime }$ are the shear and normal effective stress acting on the fault, respectively; μ is the friction coefficient ( μ=tan⁡φ ) and c is the cohesion. The CFS of the elements ${CFS}_{p}$ are the main inputs of the seismicity rate model.

### (b) Seismicity rate model

The seismicity rate is estimated by the rate-and-state friction model, which is based on the theory of earthquake nucleation [59–62]. The friction of a fault depends on the slip rate and on the state variable of re-strengthening of the fault after a slip. Unstable and seismic slip occurs on faults with rate-weakening properties when the shear strength reduces fast [61,63–65].

The number of mainshocks that are probably induced by stress variations is expressed by the relative seismicity rate R [59,66]


(2.10) $$\frac{dR}{dt}=\frac{R}{t_{c}}\left(\frac{\overset{˙}{\tau }}{\overset{˙}{\tau_{0}}}-R\right),$$

where the stressing rate is $\dot{\tau }=\frac{dCFS}{dt}$ and $\overset{˙}{\tau_{0}}$ is the initial stressing rate, $t_{c}=A\sigma /\overset{˙}{\tau_{0}}$ is the characteristic relaxation time and Aσ is a free parameter calibrated to reproduce the total number of monitored mainshocks (or expected in a given area subject to fluid injection). The ordinary differential equation (2.10) is solved using a fifth-order adaptive time step Runge–Kutta–Fehlberg algorithm using the BRUCES tool [45,67]. The absolute seismicity rate SR (the number of independent earthquakes in a certain time window) is then calculated as a function of the relative seismicity rate R and the background seismicity rate $r_{0}$ (table 2) as

**Table 2.** Parameters of the seismic models, with values for natural earthquakes and for the case of Basel EGS project.

Parameters	Natural earthquakes			Basel EGS	
SR model					
$\overset{˙}{\tau_{0}}$ (MPa/yr)	Background stressing rate			$3\times 10^{-6}$ ^a^	$10^{-7}$ for Soultz–Sous–Forêts [68]
Aσ (MPa)	Free parameter			0.09−0.25 ^a^	calibrated for each fault
$r_{0}$ (events/yr)	Background seismicity rate			0.123	[69]
GR law					
a				$a_{co}=6.08$ , $a_{post}=3.32$	[69]
b	1	[70]		$b_{co}=1.57$ , $b_{post}=1.14$	[69]
$M_{w}$	minimum value similar as the monitored			[0;3] ^a^	
ETAS model					
$K_{as}$	Free parameter			0.035 ^a^	
$c_{as}$	0.003−1.1		[71]	0.38±0.06	[69]
$p_{as}$	0.9−1.9		[71]	1.33±0.06	[69]
$\alpha_{as}$	0.3−3.1		[71]	0.8±0.06	[69]

^a^ Values are calibrated so that the models reproduce the monitored number of mainshocks (185 earthquakes with a magnitude *M_w_* > 0 [46]) and the total number of monitored earthquakes (11 200 events during and shortly after the stop of injection [8]).


(2.11) $$SR=\;R\times r_{0}.$$

The CFS is calculated for each element of the mesh according to equation (2.9). The number of independent earthquakes for each mesh element, ${SR}_{elem}$ , is estimated by multiplying the seismicity rate based on CFS by the probability that the element hosts a portion of fracture, ${p_{f}}_{elem}$ , such as


(2.12) $$SR_{elem}=\;SR\left(CFS_{elem}\right)\times p_{f_{elem}}.$$

We assume $p_{f_{elem}}=1$ for the fault elements. Because the matrix elements cannot be considered as important as the fault elements in the estimation of the seismicity rate of the domain ${SR}_{dom}$ (matrix and fault elements), we define $p_{f_{elem}}=\;p_{f}\cdot \frac{S_{elem}}{S_{max}}$ where $p_{f}$ is the probability of having a fracture in a square metre, $S_{elem}$ is the surface area of the element and $S_{max}$ is the surface area of the largest element in the mesh, equal to 10 300 m^2^.

**Figure 2.**
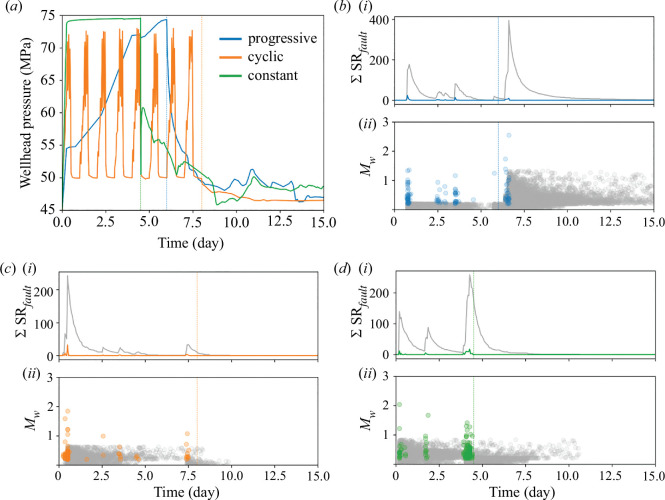
(*a*) Simulated wellhead pressure for the different injection protocols in the hydro-mechanical model. (*b*(i), *c*(i) and *d*(i)) The forecasted seismicity rate in the domain $\sum {SR}_{fault}$ through time. (*b*(ii), *c*(ii) and *d*(ii)) The estimated magnitude $M_{w}$ of the forecasted earthquakes for the progressive, cyclic and constant injection protocols, respectively. The mainshock seismicity rate and earthquakes follow the same colour code as in (*a*), while the aftershocks are in grey. Vertical lines correspond to the stop of the injections.

The seismicity rate of a fault ${SR}_{fault}$ is the sum of the ${SR}_{elem}$ of the elements of the faults. We measure the evolution of $\sum {SR}_{fault}$ to compare the seismicity rate of the discrete faulting domain of the three injection protocols (see §4 and figure 2*a*). We consider the seismicity rate of the whole domain ${SR}_{dom}$ when forecasting seismicity in two dimensions, corresponding to the sum of the seismicity rate of all the elements of the model (faults and matrix) (see §5).

The procedure described above was calibrated to reproduce the observed seismicity at Basel. A sensitivity analysis was not performed because the calibration of the model is made to estimate a total number of mainshocks and aftershocks similar to the monitored seismicity when prescribing the applied injection pressure at the Basel EGS.

### (c) Magnitude attribution with the Gutenberg-Richter law

Once the seismicity rate, i.e. the number of earthquakes for a period of time, is estimated, we use the GR law to relate the earthquake frequency to the magnitude. The GR law can be used to attribute magnitude to each forecasted earthquake [55,72]. For a specific location, it forecasts the number of earthquakes N with a magnitude greater than or equal to M according to


(2.13) logN(M)=a−b⋅M,

where a and b are constants. We adopt this stochastic law for both the mainshock and the aftershocks. We first derive a and b from the observed seismic catalogues of the study case for both co-injection and post-injection stages (table 2). The magnitude of the aftershocks, estimated by the ETAS model, is also attributed with the GR law with the maximum magnitude of the aftershock sequence set as $M=M_{i}-1.2$ , where $M_{i}$ is the mainshock magnitude [73]. Note that a and b are typically different in the injection and post-injection stages [69,74].

### (d) The Epidemic Type Aftershock Sequence model

The seismicity rate model only forecasts mainshocks, i.e. independent earthquakes. To better forecast fluid-injection induced seismicity, we adopt the ETAS model based on the Omori law [75,76]. The ETAS model estimates the aftershock sequence associated with each mainshock of magnitude $M_{i}$ , i.e. the number of aftershocks per unit of time as [56]


(2.14) $$\lambda \left(t\right)=\frac{K_{As}}{{\left(c_{As}+t-t_{i}\right)}^{p_{As}}}10^{\alpha_{As}\left(M_{i}-M_{min}\right)},$$

where $c_{As}$ is an empirical constant and $p_{As}$ is the power of the Omori law, while $K_{As}$ and $\alpha_{As}$ are parameters describing the seismic sequence (table 2). Note that this procedure does not include any spatial reference for the forecasted aftershocks; aftershocks are forecasted at the centre of the host element, at the same location as the mainshock.

## 3. Calibration and application of the hybrid method to the case of the Basel EGS

The hydro-mechanical model is designed to reproduce the fault reactivation due to fluid injection at the Basel EGS. The geometry, hydraulic and mechanical properties of the model have been defined in a previous study, the aim of which was to understand the triggering mechanisms of each fault reactivation, with special emphasis on the post-injection induced seismicity [33]. The domain consists of a seven-fault network that is based on the cluster characterisation of the monitored seismicity proposed by Deichmann *et al*. [46]. The reproduction of the reactivation of the faults is modelled on a two-dimensional horizontal domain; appropriate since most of the focal mechanisms exhibit strike-slip movement on faults with vertical dip (figure 1*a*). The location and orientation of the faults were chosen according to the orientation of the focal mechanisms and the location of the cluster swarms. Mechanical properties of the subsurface and of the initial stress conditions were inferred from different studies of the Basel EGS and/or from a calibration process (table 2) [8,47,69,77]. The monitored wellhead pressure evolution, as reported by Häring *et al*. [8], was applied as the forcing condition at the injection well of our model. Because of the two-dimensionality of the adopted model, it was not possible to reproduce the observed pressure in response to the applied injection rate [33].

The calibration of the seismicity rate model was made with the hydro-mechanical model reproducing the injection pressure response in the Basel EGS project, which lasted six days, followed by a five-hour shut-in and a bleed-off for the rest of the simulation, which stopped at day 15 (figure 1*b,c* and table 2). The initial stressing rate $\overset{˙}{\tau_{0}}$ is difficult to estimate, here we set it from an estimation made for the case of the EGS of Soultz–Sous–Forêts [68]. The free parameter Aσ of the seismicity rate model was calibrated for each fault to reproduce the total number of monitored earthquakes constituting the cluster used to design that fault. For both the design of the faulting network in the hydro-mechanical model and the calibration of the seismicity rate model, we adopt the seismic catalogue by Deichmann *et al*. [46], which reports magnitude, location and focal mechanisms for a total of 185 mainshocks with magnitude $M_{w}>0$ that were monitored during and after the hydraulic stimulation at Basel. Although our calibrated model successfully reproduces the variation of the total number of earthquakes, the trend shows some spikes compared to the more smooth curve of the observed seismicity. This is because of the adoption of a simplified network of faults derived from the clustered events at Basel within the hydro-mechanical model controlling the time evolution of the seismicity rate. In reality, each cluster is composed of several earthquakes occurring on a more complex set of faults at different close times. To assign a magnitude to the forecasted earthquakes, the parameters a and b of the GR law are assumed as estimated by Bachmann *et al*. [69] (table 2). Bachmann *et al*. [69] also confirmed that the Basel sequence follows the Omori law and used the ETAS model to forecast the elapsed time of the sequence; here we use the same values. The free parameter $K_{As}$ of the ETAS model was calibrated to estimate a total number of earthquakes (mainshock and aftershocks) equal to 11 200 events, as monitored during and shortly after the stop of injection at Basel [8]. Once all the parameters of the seismic and hydro-mechanical models are calibrated, the hybrid model is applied to forecast the seismicity under different protocols of injection.

## 4. Effects of different injection protocols on the stability of discrete faults

We simulate three injection protocols in the hydro-mechanical model of Basel EGS, successively linked to the seismic models through the CFS parameter. We use a progressive step-rate injection for six days, similar to the measured pressure at the injection wellhead of Basel EGS, but followed by a five-hour shut-in and then a bleed-off, to calibrate the models. Then, using calibrated properties (table 1) and parameters (table 2), we simulate a progressive step-rate injection, a constant injection and a cyclic injection followed by simple shut-in (figure 2*a*). To systematically compare the enhancement of permeability of faults and the induced seismicity of the three protocols, the same total volume of water was injected and the maximum applied pressure was set at 75 MPa, as the monitored maximum wellhead pressure in Basel EGS. Constant injection was applied, with the pressure set at 75 MPa for 4.5 days. The cyclic injection was based on the cyclic soft injection protocol that was applied at Pohang EGS [27], considering the long-term cycle of one day with six medium-term cycles per day. The maximum applied pressure was 75 MPa, and the stimulation lasted for eight days (figure 2*a*; note that the simulated pressure is plotted, not the one applied at the injection well in the hydro-mechanical model).

### (a) Permeability enhancement

**Figure 3.**
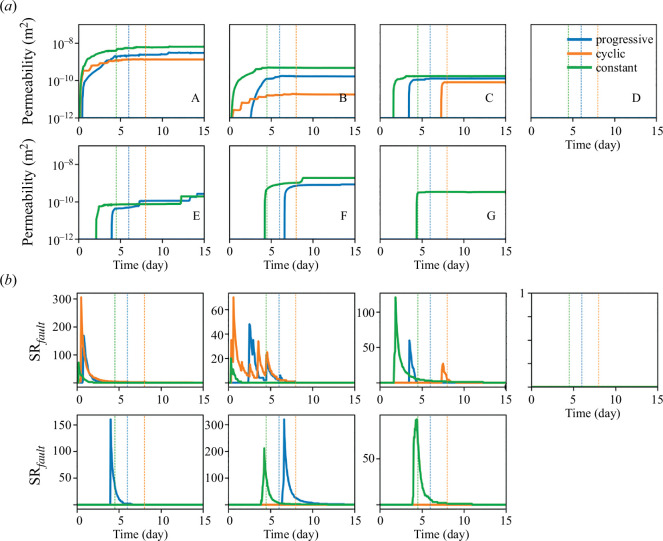
(*a*) Evolution of the permeability of the faults output from the hydro-mechanical model. The permeability varies when the fault fails in shear mode. (*b*) Seismicity rate of each fault, ${SR}_{fault},$ through time for each of the injection strategies. Although some faults reach failure conditions in the hydro-mechanical model, their CFS is too low to forecast the reactivation of the faults as seismic according to the calibrated seismicity rate model. Vertical lines correspond to the stop of the injections.

**Figure 4.**
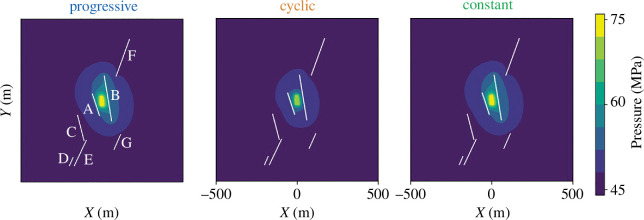
Pressure at the stop of injection for each strategy (day 6 for progressive injection, day 7.5 for cyclic injection (peak of injection of the last long-term cycle) and day 4.5 for constant injection) in the domain, outputted from the hydro-mechanical model.

As previously introduced, the aim of the hydraulic stimulation in EGS projects is to enhance the permeability of pre-existing fractures and faults in low-permeability rock. The permeability enhancement is mostly observed when faults reach shear failure conditions (figure 3*a*). The permeability of faults A, B and C increases for the three injection protocols. The permeability of farther faults (E and F) is enhanced only with the constant and the progressive injections, and the permeability of fault G only with the constant injection. The faults that are reactivated with the constant and progressive are not significantly pressurized during injection, which implies that poroelastic stress and static stress transfer are dominant triggering mechanisms of these distant faults. The effects of cyclic injection are limited to the faults in the vicinity of the injection well. In this protocol, the pressure buildup and consequent mechanisms are insufficient to activate shear failure on the distant faults (figure 4). Therefore, the constant and the progressive injection protocols similarly enhance the permeability of the discrete faults, while the cyclic protocol leads to a limited permeability enhancement. The delay in fault permeability enhancement of approximately two days between the constant and progressive protocols was caused by the time difference in the diffusion of the pressure sufficient to enhance the permeability of faults in the domain—the progressive injection protocol injects at low pressures at early times. Interestingly, the reactivation and permeability enhancement of fault F during progressive injection is the unique reactivation that occurs after the stop of injection in all the cases.

### (b) Forecasted seismicity

The hybrid method based on the hydro-mechanical simulations forecasts the mainshocks and aftershocks for the three injections. Progressive injection induces the most mainshocks, due to the reactivation of the faults A, B, C, E and F. Fault F reactivation occurs after the stop of injection and translates into a large peak of the seismicity rate of the faults $\sum {SR}_{fault}$ for the case of the progressive injection. Constant injection, which reactivates similar faults (A, B, C, F and G) during injection to those of the progressive injection has, nonetheless, smaller peaks of the seismicity rate ${SR}_{fault}$ for most faults (figure 2*b,d* and figure 3*b*). Cyclic injection induces the largest peak of the seismicity rate of the faults $\sum {SR}_{fault}$ at the start of the stimulation, when faults A and B reactivate simultaneously, and their permeability is enhanced (figures 2*c* and 3*b*).

**Figure 5.**
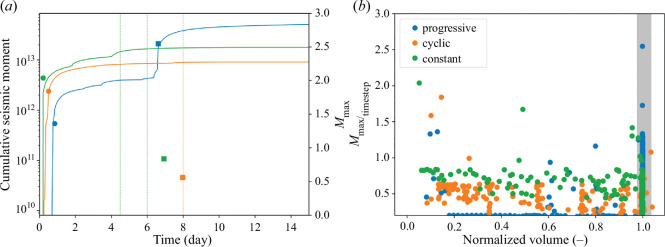
(*a*) Cumulative seismic moment $M_{o}=\;10^{1.5\;M_{w}\;+\;6.07\;}$ [78] of the forecasted earthquakes (lines) and maximum magnitude $M_{max}$ for both co-injection (circle) and post-injection (square) stages. Vertical lines correspond to the stop of injections. (*b*) Maximum earthquake magnitude as a function of the normalized injected volume for each timestep. The grey band corresponds to the time after the stop of injection.

In our simulations, the magnitude of forecasted seismic events does not reach the magnitude of the monitored seismicity of the Basel EGS. Nonetheless, the trend of the magnitude of the events in the progressive injection is similar to the Basel EGS, with the largest event occurring after the stop of injection (figure 2*b*). The seismicity rate indicates in all cases the destabilization of the reservoir. The constant and cyclic protocols induce seismicity from the start of injection, while the onset of seismicity in the progressive injection is delayed for a few hours (figures 2 and 5*a*). Despite the progressive injection presenting the largest total number of mainshocks (progressive: 147, cyclic: 64 and constant: 111 mainshocks), its cumulative seismic moment is the lowest, presenting steps caused by the different timings of fault reactivations (figure 5*a*). Interestingly, the cumulative seismic moment at the end of the stimulation is similar for the cyclic and constant injections (figure 5*a*), but the permeability enhancement is much lower in the cyclic than in the constant injection (figure 3*a*). It is worth noting that there is no correlation between the injected volume and the forecasted magnitude, and that the maximum magnitude $M_{max}$ is induced towards the beginning of injection in the three protocols (figure 5*b*). This result questions relationships that estimate the maximum expected magnitude as a function of the injected volume (e.g. [79]) and highlights the importance of triggering mechanisms other than pore pressure changes [33,80].

As far as post-injection seismicity is concerned, the progressive injection strategy induces the largest magnitude earthquake at the stop of injection, as it was in the case of the Basel EGS (figures 2*b* and 5*a*). This large event was due to the reactivation and permeability enhancement of fault F. Cyclic and constant injections do not induce mainshocks after the stop of injection, but the co-injection largest-magnitude earthquake occurs at the beginning of the stimulation with a similar magnitude to that of the progressive post-injection earthquake (figure 5*a*). The mainshocks forecasted after the end of injection in the progressive injection lead to a significant aftershock sequence.

In summary, different protocols injecting the same volume of water show different responses of the subsurface. Cyclic injection has limited effect in enhancing fault permeability. Progressive injection delays seismicity and causes an accumulation of stresses at the faults that induce larger stress drops, inducing larger peaks of ${SR}_{fault}$ and $\sum {SR}_{fault}$ , meaning a greater risk of high-magnitude earthquake nucleation. Progressive injection is also the only scenario with post-injection simulated mainshocks. The best strategy seems to be constant injection, which reactivates the faults from the start, enhancing permeability in most faults, and limits the post-injection seismicity on pre-existing faults.

## 5. Spatially forecasting the seismicity

**Figure 6.**
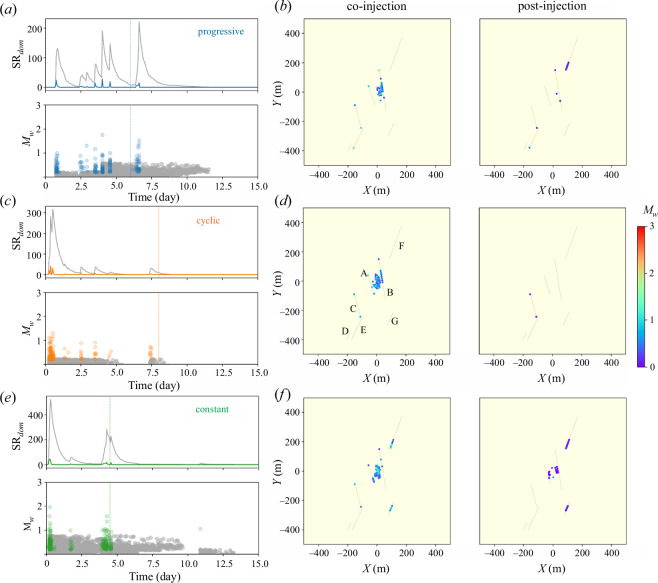
Time evolution of forecasted seismicity rate of the whole domain ${SR}_{dom}$ and corresponding magnitude for the (*a*) progressive, (*c*) cyclic and (*e*) constant injections. Colours represent the mainshock seismicity rate and magnitude, while grey represents the aftershocks. (*b*, *d* and *f*) The two-dimensional spatial distribution of the co- and the post-injection seismicity plotted for the three protocols. The colour scale represents the magnitude of the estimated earthquakes. For plotting purpose, the location of the earthquakes within the faults and in the matrix is adjusted to avoid overlapping.


The proposed hybrid methodology can be used to spatially forecast the seismicity rate in the whole domain ${SR}_{dom}$ , including the matrix elements (figure 6). For the matrix, we set Aσ equal to 0.25 , the largest value for the fault calibration. We assume a spatial distribution of the probability of having a fracture in a square metre, $p_{f}$ , to better represent the presence of conjugate fractures in the vicinity of the well [77]. We consider $p_{f}=u\times P21$ to calibrate the forecast of approximately 50 mainshocks in the matrix elements, where $u=3\times 10^{-6}$ and P21 is the areal fracture intensity, which expresses the length of fracture traces per unit area [81]. P21 is estimated by field observations of the linear fracture intensity, expressed by the number of fracture traces per unit length P10 . Because the mean length of fracture traces is equal to the borehole diameter divided by the cosine of the fracture dip, 70° in the study case of the Basel EGS, the areal fracture intensity is given by

(5.1) $$P21=P10\;/cos\left(70^{\circ }\right).$$

For the Basel EGS, values of ${P10}_{low}=0.25$ m^−1^ and ${P10}_{high}=0.95$ m^−1^ have been estimated for the least and the most intensely fractured zones, respectively [8]. However, we set P10 of the matrix elements between 0.10 and 0.25 m^−1^ to forecast a coherent number of mainshocks, less than 250 mainshocks in total, in both fault and matrix elements (figure 1*a*). To estimate the CFS rate of the matrix elements, we randomly assign to each of the mesh elements a fracture orientation of N160 or N40, which corresponds to the main conjugate faults with the probability of 0.6 and 0.4, respectively [77]. The seismicity rate of the whole domain ${SR}_{dom}$ is similar to the seismicity rate of the domain $\sum {SR}_{fault}$ (calculated from the discrete faults only) (figure 2*b,c,d* and 6*a,c,e*), but the two-dimensional forecasting allows stimulation of the potential induced seismicity in different areas of the reservoir without mapped faults (figure 6). With the forecasting of seismicity in the matrix, the constant injection induces a mainshock late after the stop of injection, probably caused by post-injection pore pressure diffusion.

## 6. Discussion

The proposed hybrid methodology allows us to estimate the seismicity nucleation induced by different injection protocols. The implementation of the seismicity rate and the ETAS models combined with a numerical model is efficient in forecasting fluid-injection induced seismicity. Hydro-mechanical models simulate the pressure and stress variations due to fluid injection and extraction in hot rock. In cases of discrete faults, it can also simulate stress redistribution from fault reactivations. The use of numerical models provides the possibility to understand and reproduce the complex and coupled triggering processes (e.g. pressure-driven processes, thermal interactions and mechanical behaviours) and the possibility to investigate their mitigation. Our model is limited to the hydro-mechanical problem caused by the negligible effect of thermal coupling as cooling does not significantly propagate away from the injection well during the short duration of the stimulation [82]. Our discrete-fault hydro-mechanical model of the case of the Basel EGS is, however, complex enough to forecast seismicity induced by hydraulic stimulation as both poromechanical stresses and static stress transfer are simulated in addition to pore pressure diffusion. We are optimistic that models will be developed to solve the coupled thermo-hydro-mechanical-chemical problems faster and more efficiently in the future with the development of the hHigh-pPerformance cComputing, ebabling us to forecast induced seismicity both during hydraulic stimulation and operation of EGS.

One of the weaknesses of the hybrid methodology is the estimation of large-magnitude earthquakes. Because the GR law depends on the number of seismic events, the model does not estimate magnitudes as large as the monitored earthquakes in the Basel EGS. We favoured the calibration of the seismicity rate rather than the calibration of the magnitude attribution model. Moreover, the GR law attributes stochastically the magnitude, which can affect the forecasting of the aftershocks via the ETAS model. It also affects the interpretation of the cumulative seismic moment. The second limitation of the methodology is that the ETAS model is used here to attribute an aftershock sequence to each mainshock without spatial distribution. This aspect affects the two-dimensional spatial forecasting, but more importantly, the effects of stress drop that differ with the location of the nucleation of each earthquake, mainshocks and aftershocks. Moreover, the construction of the model was possible thanks to seismic interpretations *posterior* to the injection itself. In the case of the development of new EGS projects, the fault network is usually not yet known. Yet, a simple fault zone could already be sufficient to initiate the model [20]. This hybrid methodology could be associated with a real-time forecasting model that could update the simplified fault network as a function of real-time seismic interpretation, using the same dynamics as the Adaptive Traffic Light System adjustments with real-time monitoring [35,40].

Co-injection induced seismicity is partly controlled by the injection parameters. The comparison between three simple protocols (progressive, constant and cyclic injections) shows their different effects on the stability of the subsurface. Cyclic injection, designed on the cyclic soft stimulation performed at the Pohang EGS [27], results in a limited pore pressure buildup, as the periods with low-pressure injection cause pressure drop in the whole domain. The limited pressure buildup in the domain restricts fault reactivation to the vicinity of the injection well. Cyclic injection, investigated with the proposed methodology, does not significantly enhance the permeability of the faults, and relatively large-magnitude induced seismicity is not prevented, two decisive objectives for economic viability and public perception of EGS projects. On the other hand, constant and progressive injections seem to be similar in enhancing fault permeability, with a time difference due to the time required to inject the same volume. The main difference between the two is that constant injection disturbs the stress conditions abruptly at the start of the injection, which induces the peak of seismicity early in the stimulation. Progressive injection in our model has a larger peak due to the simultaneous reactivations of two large faults. In addition, the reactivations of the faults induce larger stress drops than in the case of the constant injection. Constant injection accumulates less stresses on the faults and for a shorter period of time, which mitigates large seismicity rates and, therefore, large-magnitude earthquakes. Because the seismicity rate is sensitive to abrupt stress changes, constant injection induces less forecasted seismicity through smaller pressure changes during the stimulation. Moreover, the duration of injection affects the evolution of aseismic slip, and a shorter injection duration limits the duration of the post-injection aseismic slip propagation [83]. With all these arguments, constant injection seems to be the most promising stimulation protocol.

## 7. Conclusion

EGS need to be deployed to facilitate the low-carbon energy transition. The enhancement of permeability and the mitigation of co- and post-injection induced seismicity are the current principal challenges to improve the public perception and the economic viability of EGS projects. Constant injection seems to be the most efficient strategy, based on the comparison with cyclic and progressive (with the same injected volume) injections, to both enhance the permeability of pre-existing faults and control the induced seismicity. Its short duration limits the stress accumulation on faults and the post-injection aseismic slip. Cyclic injection, which is one of the most recently developed protocols, based on our hybrid method, does not enhance the permeability of the discrete faults outside the vicinity of the injection well. The hybrid model, based on a strong and complex coupled hydro-mechanical model associated with the seismicity rate and aftershock-sequence models, enables the simulation of different known triggering mechanisms of induced seismicity and provides freedom to simulate different injection protocols. Its coupling with a real-time monitoring to adjust the fault network and mechanical parameters could help to forecast and to mitigate the seismicity during the hydraulic stimulations and production stages of EGS projects.

## Data Availability

This article has no additional data.

## References

[1] IPCC . 2023 Summary for Policymakers. In: Climate Change 2023: Synthesis Report. Contribution of Working Groups I, II and III to the Sixth Assessment Report of the Intergovernmental Panel on Climate Change [Core Writing Team, H. Lee and J. Romero (eds.)]. pp. 1–34. IPCC. (10.59327/IPCC/AR6-9789291691647.001)

[2] Tester JW *et al* . 2007 Impact of enhanced geothermal systems on US energy supply in the twenty-first century. Phil. Trans. R. Soc. A **365** , 1057–1094. (10.1098/rsta.2006.1964) 17272236

[3] Buijze L *et al* . 2019 Review of induced seismicity in geothermal systems worldwide and implications for geothermal systems in the Netherlands. Neth. J. Geosci. **98** , e13. (10.1017/njg.2019.6)

[4] Evans KF , Zappone A , Kraft T , Deichmann N , Moia F . 2012 A survey of the induced seismic responses to fluid injection in geothermal and CO2 reservoirs in Europe. Geothermics **41** , 30–54. (10.1016/j.geothermics.2011.08.002)

[5] Majer EL , Baria R , Stark M , Oates S , Bommer J , Smith B , Asanuma H . 2007 Induced seismicity associated with enhanced geothermal systems. Geothermics **36** , 185–222. (10.1016/j.geothermics.2007.03.003)

[6] Pine RJ , Batchelor AS . 1984 Downward migration of shearing in jointed rock during hydraulic injections. Int. J. Rock Mech. Min. Sci. **21** , 249–263. (10.1016/0148-9062(84)92681-0)

[7] Zang A , Oye V , Jousset P , Deichmann N , Gritto R , McGarr A , Majer E , Bruhn D . 2014 Analysis of induced seismicity in geothermal reservoirs – an overview. Geothermics **52** , 6–21. (10.1016/j.geothermics.2014.06.005)

[8] Häring MO , Schanz U , Ladner F , Dyer BC . 2008 Characterisation of the Basel 1 enhanced geothermal system. Geothermics **37** , 469–495. (10.1016/j.geothermics.2008.06.002)

[9] Ellsworth WL , Giardini D , Townend J , Ge S , Shimamoto T . 2019 Triggering of the Pohang, Korea, Earthquake (Mw 5.5) by Enhanced Geothermal System Stimulation. Seismological Research Letters **90(5)** , 1844–1858. (10.1785/0220190102)

[10] Grigoli F , *et al* . 2018 The november 2017 M _w_ 5.5 Pohang earthquake: a possible case of induced seismicity in South Korea. Science **360** , 1003–1006. (10.1126/science.aat2010) 29700226

[11] Kim KH , Ree JH , Kim Y , Kim S , Kang SY , Seo W . 2018 Assessing whether the 2017 M w 5.4 Pohang earthquake in South Korea was an induced event. Science **360** , 1007–1009. (10.1126/science.aat6081) 29700224

[12] Fiori R , Vergne J , Schmittbuhl J , Zigone D . 2023 Monitoring induced microseismicity in an urban context using very small seismic arrays: the case study of the Vendenheim EGS project. GEOPHYSICS **88** , WB71–WB87. (10.1190/geo2022-0620.1)

[13] Lengliné O *et al* . 2023 The largest induced earthquakes during the GEOVEN deep geothermal project, Strasbourg, 2018–2022: from source parameters to intensity maps. Geophys. J. Int. **234** , 2445–2457. (10.1093/gji/ggad255)

[14] Schmittbuhl J , Lambotte S , Lengliné O , Grunberg M , Jund H , Vergne J , Cornet F , Doubre C , Masson F . 2022 Induced and triggered seismicity below the city of Strasbourg. C. R. - Geosci **353** , 561–584. (10.5802/crgeos.71)

[15] Ellsworth WL . 2013 Injection-induced earthquakes. Science **341** , 1225942. (10.1126/science.1225942) 23846903

[16] Ge S , Saar MO . 2022 Review: Induced seismicity during geoenergy development—a hydromechanical perspective. J. Geophys. Res. **127** . (10.1029/2021JB023141)

[17] Cornet FH . 2012 The relationship between seismic and aseismic motions induced by forced fluid injections. Hydrogeol. J. **20** , 1463–1466. (10.1007/s10040-012-0901-z)

[18] Guglielmi Y , Cappa F , Avouac JP , Henry P , Elsworth D . 2015 Seismicity triggered by fluid injection-induced aseismic slip. Science **348** , 1224–1226. (10.1126/science.aab0476) 26068845

[19] Wei S *et al* . 2015 The 2012 Brawley swarm triggered by injection-induced aseismic slip. Earth Planet. Sci. Lett. **422** , 115–125. (10.1016/j.epsl.2015.03.054)

[20] Boyet A , De Simone S , Vilarrasa V . 2023 Physics-based modeling to understand and to propose forecasting methods of induced seismicity. Seismol. Res. Lett. **94** , 2666–2678. (10.1785/0220230109)

[21] Yoon JS , Zang A , Stephansson O . 2014 Numerical investigation on optimized stimulation of intact and naturally fractured deep geothermal reservoirs using hydro-mechanical coupled discrete particles joints model. Geothermics **52** , 165–184. (10.1016/j.geothermics.2014.01.009)

[22] Yoon JS , Zimmermann G , Zang A . 2015 Discrete element modeling of cyclic rate fluid injection at multiple locations in naturally fractured reservoirs. Int. J. Rock Mech. Min. Sci. **74** , 15–23. (10.1016/j.ijrmms.2014.12.003)

[23] Ji Y , Fang Z , Wu W . 2021 Fluid overpressurization of rock fractures: Experimental investigation and analytical modeling. Rock Mech. Rock Eng. **54** , 3039–3050. (10.1007/s00603-021-02453-8)

[24] Ji Y , Yoon JS , Zang A , Wu W . 2021 Mitigation of injection-induced seismicity on undrained faults in granite using cyclic fluid injection: a laboratory study. Int. J. Rock Mech. Min. Sci. **146** , 104881. (10.1016/j.ijrmms.2021.104881)

[25] Zhu JB , Kang JQ , Elsworth D , Xie HP , Ju Y , Zhao J . 2021 Controlling induced earthquake magnitude by cycled fluid injection. Geophys. Res. Lett. **48** . (10.1029/2021GL092885)

[26] Noël C , Passelègue FX , Giorgetti C , Violay M . 2019 Fault reactivation during fluid pressure oscillations: Transition from stable to unstable slip. J. Geophys. Res. **124** , 10940–10953. (10.1029/2019JB018517)

[27] Hofmann H *et al* . 2019 First field application of cyclic soft stimulation at the Pohang Enhanced Geothermal System site in Korea. Geophys. J. Int. **217** , 926–949. (10.1093/gji/ggz058)

[28] Kwiatek G *et al* . 2019 Controlling fluid-induced seismicity during a 6.1-km-deep geothermal stimulation in Finland. Sci. Adv. **5** , eaav7224. (10.1126/sciadv.aav7224) 31049397 PMC6494490

[29] Zang A , Niemz P , von Specht S , Zimmermann G , Milkereit C , Plenkers K , Klee G . 2023 Comprehensive data set of in-situ Hydraulic stimulation experiments for geothermal purposes at the Äspö hard rock laboratory (Sweden). ESSD – Land/Geophysics and geodesy. (10.5194/essd-2023-170)

[30] Zang A , Zimmermann G , Hofmann H , Stephansson O , Min KB , Kim KY . 2019 How to reduce fluid-injection-induced seismicity. Rock Mech. Rock Eng. **52** , 475–493. (10.1007/s00603-018-1467-4)

[31] Alghannam M , Juanes R . 2020 Understanding rate effects in injection-induced earthquakes. Nat. Commun. **11** , 3053. (10.1038/s41467-020-16860-y) 32546793 PMC7298001

[32] McClure MW , Horne RN . 2011 Investigation of injection-induced seismicity using a coupled fluid flow and rate/state friction model. GEOPHYSICS **76** , WC181–WC198. (10.1190/geo2011-0064.1)

[33] Boyet A , De Simone S , Ge S , Vilarrasa V . 2023 Poroelastic stress relaxation, slip stress transfer and friction weakening controlled post-injection seismicity at the Basel Enhanced Geothermal System. Commun. Earth Environ. **4** , 1–13. (10.1038/s43247-023-00764-y)37325084

[34] Broccardo M , Mignan A , Wiemer S , Stojadinovic B , Giardini D . 2017 Hierarchical bayesian modeling of fluid‐induced seismicity. Geophys. Res. Lett. **44** , 11. (10.1002/2017GL075251)

[35] Ritz VA , Mizrahi L , Repollés VC , Rinaldi AP , Hjörleifsdóttir V , Wiemer S . 2023 Pseudo-prospective forecasting of induced and natural seismicity in the hengill geothermal field. Preprints. (10.22541/essoar.168500354.49240043/v1)

[36] Shapiro SA , Dinske C , Kummerow J . 2007 Probability of a given‐magnitude earthquake induced by a fluid injection. Geophys. Res. Lett. **34** , L22314. (10.1029/2007GL031615)

[37] Shapiro SA , Dinske C , Langenbruch C , Wenzel F . 2010 Seismogenic index and magnitude probability of earthquakes induced during reservoir fluid stimulations. Lead. Edge **29** , 304–309. (10.1190/1.3353727)

[38] Shapiro SA . 2018 Seismogenic index of underground fluid injections and productions. J. Geophys. Res. **123** , 7983–7997. (10.1029/2018JB015850)

[39] Mignan A , Broccardo M , Wang Z . 2021 Comprehensive survey of seismic hazard at geothermal sites by a meta-analysis of the underground feedback activation parameter afb. Energies **14** , 7998. (10.3390/en14237998)

[40] Mignan A , Broccardo M , Wiemer S , Giardini D . 2017 Induced seismicity closed-form traffic light system for actuarial decision-making during deep fluid injections. Sci. Rep. **7** , 13607. (10.1038/s41598-017-13585-9) 29051528 PMC5648767

[41] Clasen Repolles V , Rinaldi AP , Ciardo F , Passarelli L , Wiemer S , Bedretto Team . 2023 Performance comparison of newly developed hydro-mechanical (hybrid) models for real-time induced seismicity forecasting (10.5194/egusphere-egu23-12783)

[42] Dempsey D , Riffault J . 2019 Response of induced seismicity to injection rate reduction: models of delay, Decay, Quiescence, recovery, and Oklahoma. Water Resour. Res. **55** , 656–681. (10.1029/2018WR023587)

[43] Goertz-Allmann BP , Wiemer S . 2013 Geomechanical modeling of induced seismicity source parameters and implications for seismic hazard assessment. Geophysics **78** , KS25–KS39. (10.1190/geo2012-0102.1)

[44] Karvounis D , Wiemer S . 2022 A discrete fracture hybrid model for forecasting diffusion-induced seismicity and power generation in enhanced geothermal systems. Geophys. J. Int. **230** , 84–113. (10.1093/gji/ggac056)

[45] Luu K , Schoenball M , Oldenburg CM , Rutqvist J . 2022 Coupled Hydromechanical Modeling of Induced Seismicity From CO _2_ Injection in the Illinois Basin. J. Geophys. Res. **127** , 1–19. (10.1029/2021JB023496)

[46] Deichmann N , Kraft T , Evans KF . 2014 Identification of faults activated during the stimulation of the Basel geothermal project from cluster analysis and focal mechanisms of the larger magnitude events. Geothermics **52** , 84–97. (10.1016/j.geothermics.2014.04.001)

[47] Herrmann M , Kraft T , Tormann T , Scarabello L , Wiemer S . 2019 A consistent high‐resolution catalog of induced seismicity in basel based on matched filter detection and tailored post‐processing. J. Geophys. Res. **124** , 8449–8477. (10.1029/2019JB017468)

[48] Kraft T , Deichmann N . 2014 High-precision relocation and focal mechanism of the injection-induced seismicity at the Basel EGS. Geothermics **52** , 59–73. (10.1016/j.geothermics.2014.05.014)

[49] Bachmann CE , Wiemer S , Goertz-Allmann BP , Woessner J . 2012 Influence of pore-pressure on the event-size distribution of induced earthquakes: Pore pressure and earthquake size distribution. Geophys. Res. Lett. **39** , 1–7. (10.1029/2012GL051480)

[50] Terakawa T . 2014 Evolution of pore fluid pressures in a stimulated geothermal reservoir inferred from earthquake focal mechanisms. Geophys. Res. Lett. **41** , 7468–7476. (10.1002/2014GL061908)

[51] Mukuhira Y , Dinske C , Asanuma H , Ito T , Häring MO . 2017 Pore pressure behavior at the shut‐in phase and causality of large induced seismicity at Basel, Switzerland. J. Geophys. Res. **122** , 411–435. (10.1002/2016JB013338)

[52] Catalli F , Meier MA , Wiemer S . 2013 The role of Coulomb stress changes for injection‐induced seismicity: The Basel enhanced geothermal system. Geophys. Res. Lett. **40** , 72–77. (10.1029/2012GL054147)

[53] Catalli F , Rinaldi AP , Gischig V , Nespoli M , Wiemer S . 2016 The importance of earthquake interactions for injection‐induced seismicity: retrospective modeling of the basel enhanced geothermal system. Geophys. Res. Lett. **43** , 4992–4999. (10.1002/2016GL068932)

[54] Andrés S , Santillán D , Mosquera JC , Cueto-Felgueroso L . 2019 Thermo-poroelastic analysis of induced seismicity at the basel enhanced geothermal system. Sustainability **11** , 6904. (10.3390/su11246904)

[55] Gutenberg B , Richter CF . 1942 Earthquake magnitude, intensity, energy, and acceleration*. Seismol. Soc. Am. Bull. **32** , 163–191. (10.1785/BSSA0320030163)

[56] Ogata Y . 1988 Statistical models for earthquake occurrences and residual analysis for point processes. J. Am. Stat. Assoc. **83** , 9–27. (10.1080/01621459.1988.10478560)

[57] Olivella S , Gens A , Carrera J , Alonso EE . 1996 Numerical formulation for a simulator (CODE_BRIGHT) for the coupled analysis of saline media. Eng. Comput. **13** , 87–112. (10.1108/02644409610151575)

[58] Olivella S , Alonso EE . 2008 Gas flow through clay barriers. Géotechnique **58** , 157–176. (10.1680/geot.2008.58.3.157)

[59] Dieterich J . 1994 A constitutive law for rate of earthquake production and its application to earthquake clustering. J. Geophys. Res **99** , 2601–2618. (10.1029/93JB02581)

[60] Meyer H , Smith JD , Bourne S , Avouac JP . 2023 An integrated framework for surface deformation modelling and induced seismicity forecasting due to reservoir operations. Geol. Soc. Spec. Publ. **528** , 299–318. (10.1144/SP528-2022-169)

[61] Scholz CH . 1998 Earthquakes and friction laws. Nature **391** , 37–42. (10.1038/34097)

[62] Scholz CH . 2002 The mechanics of earthquakes and Faulting. Cambridge: Cambridge University Press. (10.1017/CBO9780511818516) 17734719

[63] Dieterich JH . 1978 Time-dependent friction and the mechanics of stick-slip. Pure and Applied Geophysics **116** , 790–806. (10.1007/BF00876539)

[64] Marone C . 1998 The effect of loading rate on static friction and the rate of fault healing during the earthquake cycle. Nature **391** , 69–72. (10.1038/34157)

[65] Ruina A . 1983 Slip instability and state variable friction laws. J. Geophys. Res. **88** , 10359–10370. (10.1029/JB088iB12p10359)

[66] Segall P , Lu S . 2015 Injection‐induced seismicity: poroelastic and earthquake nucleation effects. J. Geophys. Res. **120** , 5082–5103. (10.1002/2015JB012060)

[67] Luu K . 2022 Bruces: a bunch of really useful codes for earthquake stuff (v0.5.0) (10.5281/ZENODO.6422572)

[68] Hossein Hakimhashemi A , Schoenball M , Heidbach O , Zang A , Grünthal G . 2014 Forward modelling of seismicity rate changes in georeservoirs with a hybrid geomechanical–statistical prototype model. Geothermics **52** , 185–194. (10.1016/j.geothermics.2014.01.001)

[69] Bachmann CE , Wiemer S , Woessner J , Hainzl S . 2011 Statistical analysis of the induced Basel 2006 earthquake sequence: introducing a probability-based monitoring approach for enhanced geothermal systems. Geophys. J. Int. **186** , 793–807. (10.1111/j.1365-246X.2011.05068.x)

[70] Wiemer S , Wyss M . 2002 Mapping spatial variability of the frequency-magnitude distribution of earthquakes. Advances in Geophysics **45** , 259–302. (10.1016/S0065-2687(02)80007-3)

[71] Ogata Y . 1992 Detection of precursory relative quiescence before great earthquakes through a statistical model. J. Geophys. Res. **97** , 19845–19871, (10.1029/92JB00708)

[72] Navas-Portella V , Jiménez A , Corral Á . 2020 No significant effect of coulomb stress on the gutenberg-richter law after the landers earthquake. Sci. Rep. **10** , 2901. (10.1038/s41598-020-59416-2) 32075986 PMC7031507

[73] Båth M . 1965 Lateral inhomogeneities of the upper mantle. Tectonophysics **2** , 483–514. (10.1016/0040-1951(65)90003-X)

[74] Ruiz-Barajas S , Sharma N , Convertito V , Zollo A , Benito B . 2017 Temporal evolution of a seismic sequence induced by a gas injection in the Eastern coast of Spain. Sci. Rep. **7** , 2901. (10.1038/s41598-017-02773-2) 28588269 PMC5460170

[75] Omori F . 1894 On after-shocks. J. Seismol. Soc. Jpn. 71–80.

[76] Utsu T . 1961 A statistical study on the occurrence of aftershocks. Geophys. Mag. **30** , 521–605.

[77] Mukuhira Y , Asanuma H , Niitsuma H , Häring MO . 2013 Characteristics of large-magnitude microseismic events recorded during and after stimulation of a geothermal reservoir at Basel, Switzerland. Geothermics **45** , 1–17. (10.1016/j.geothermics.2012.07.005)

[78] Kanamori H , Brodsky EE . 2004 The physics of earthquakes. Rep. Prog. Phys. **67** , 1429–1496. (10.1088/0034-4885/67/8/R03)

[79] McGarr A . 2014 Maximum magnitude earthquakes induced by fluid injection: limits on fluid injection earthquakes. J. Geophys. Res. Solid Earth **119** , 1008–1019. (10.1002/2013JB010597)

[80] Vilarrasa V , De Simone S , Carrera J , Villaseñor A . 2021 Unraveling the Causes of the Seismicity Induced by Underground Gas Storage at Castor, Spain. Geophys. Res. Lett. **48** , 1–10. (10.1029/2020GL092038)

[81] Dershowitz WS , Herda HH . 1992 Interpretation of fracture spacing and intensity. In The 33rd US Symposium on Rock Mechanics (USRMS).

[82] De Simone S , Vilarrasa V , Carrera J , Alcolea A , Meier P . 2013 Thermal coupling may control mechanical stability of geothermal reservoirs during cold water injection. Phys. Chem. Earth, Parts A/B/C **64** , 117–126. (10.1016/j.pce.2013.01.001)

[83] Jacquey AB , Viesca RC . 2023 Nucleation and Arrest of Fluid‐Induced Aseismic Slip. Geophys. Res. Lett. **50** , 1–10. (10.1029/2022GL101228)

